# Epitope mapping of recombinant *Salmonella enterica* serotype Heidelberg flagellar hook-associated protein by in silico and in vivo approaches

**DOI:** 10.1186/s12917-025-04479-4

**Published:** 2025-02-06

**Authors:** Hung-Yueh Yeh

**Affiliations:** United States Department of Agriculture, Agricultural Research Service, U.S. National Poultry Research Center, 950 College Station Road, Athens, GA 30605-2720 USA

**Keywords:** *Salmonella*, FlgK protein, Flagellar hook-associated protein, Chicken, Epitope mapping, Immunoinformatics

## Abstract

**Background:**

*Salmonella* is a leading cause of human acute bacterial gastroenteritis worldwide. Outbreaks of human salmonellosis have often been associated with consumption of contaminated poultry products. Various strategies have been explored to control this microorganism during poultry production and processing. Vaccination of broiler chickens is regarded as one of the effectives means to control this microorganism. The aim of the present study was to compare the epitope identification in the *Salmonella enterica* serotype Heidelberg FlgK protein by in silico prediction and in vivo experiment with mass spectrometry in association with immunoprecipitation proteomics.

**Results:**

The *Salmonella* serotype Heidelberg FlgK protein contains 553 amino acids with a molecular mass of 61 kDa. This protein is conserved among *Salmonella* serotype Heidelberg isolates. The results show that both approaches identified three common shared consensus peptide epitope sequences at the positions of 77–95, 243–255 and 358–373 in the *Salmonella* serotype Heidelberg FlgK protein.

**Conclusions:**

These findings provide a rational for further evaluation of these shared linear epitopes in vaccine development to cover the chicken population.

## Introduction

The genus *Salmonella* has two species: *S. enterica* and *S. bongori*. The latter is a commensal found in cold-blooded animals [63]. *S. enterica *is further classified into six subspecies [13]. Among subspecies, *S. enterica* subsp. *enterica *is comprised of more than 2,500 serotypes based on the Kauffmann-White scheme [34]. In addition, *S. enterica* subsp. *enterica* has often been broadly classified into two groups: typhoidal *Salmonella* serovar (*S.* Typhi and *S.* Paratyphi) and non-typhoidal *Salmonella* serovar (e.g., *S.* Typhimurium, *S.* Enteriditis, *S. *Infantis etc.) [30, 45]. Non-typhoidal *Salmonella *is a leading bacterial pathogen that causes human foodborne illnesses around the globe [14, 23, 39, 67, 35]. Consumption and handling of contaminated poultry meat and eggs are regarded as a major source for human salmonellosis [5, 20, 25, 57, 68, 78]. Outbreaks of salmonellosis result in huge economic losses to poultry industry and impact on public health to communities [49, 73]. The studies have demonstrated that except for two *Salmonella *serotypes (poultry-adapted pathogens) [7, 24], other *Salmonella *serotypes are considered as members of the normal microbiome in chicken gastrointestinal tract [41, 48, 54, 70]. To mitigate or eliminate *Salmonella *contamination of food products from poultry, many strategies have been investigated, including but not limited to hygiene and biosecurity measures, use of alternatives to antibiotics and addition of feed additives [62, 74]. Because *S. *Heidelberg has been found predominantly in poultry production worldwide over the years [12], this serotype was selected for analysis in this study. We are interested in vaccine development for controlling *Salmonella* during live broiler production. The selection of *Salmonella* targets as antigens that can induce host strong and protective immune response is critical to have the successful vaccine development.

Bacterial flagellum is a long hairy motile organelle that helps microorganisms to move toward the favorable and away from the detrimental surroundings [3, 46, 53]. A flagellum can be divided into three distinguished structural regions: (1) a basal body anchored in the outer and inner membranes, (2) a flexible hook connected between a basal body and a filament, and (3) a helical filament involved in adhesion and invasion [3, 46, 53]. A complete flagellum is composed of more than 40 proteins. Among them, we are interested in the flagellar hook-associated protein 1 (FlgK). This protein is not only required for proper flagellar filament formation [33, 42], but also is an important virulence factor in pathogenesis [6, 27, 66, 69, 80, 81] and a target for host immune responses [43]. Our preliminary study demonstrated that the anti-FlgK antibodies were prevalent in the poultry flock [83], suggesting that this protein may be used for the vaccine development for broilers to control *Salmonella*.

The current advances in nucleotide sequencing technology and immunoinformatics have revolutionized vaccine development during emergence, re-emergence and dissemination of resistant microorganisms [75]. The use of the genetic information of the pathogen for the potential vaccine development has been named “reverse vaccinology” [61]. The process for designing vaccines with the reverse vaccinology approach includes (1) retrieving the pathogen genome from the public databases, (2) analyzing the sequences with various immunoinformatic analysis tools to identify complete repertoires of the specific proteins or epitopes, (3) cloning, expressing and purifying recombinant proteins identified above, and (4) screening these proteins through high-throughput vaccination in animals to determine the potential for vaccines [61, 75]. The advantages of these types of vaccine development are not only cost- and time-effective and maximal efficacy, but also minimize adverse reactions [8, 71]. The vaccine against *Neisseria meningitidis* serotype B was the first successful case of using the reverse vaccinology approach [50]. Further, because of the successful mRNA vaccine technology for COVID-19, the use of mRNA encoding the desired proteins/epitopes may replace the traditional peptides/proteins vaccines.

In *Salmonella*, several recent studies applied immunoinformatic tools to design multi-epitope vaccines [1, 4, 8, 71], but these targets have never been tested in animals. The aims of this study were to (1) use immunoinformatic tools to analyze and predict the potential B-lymphocyte epitopes from the *Salmonella* serovar Heidelberg (FlgK) protein, and (2) compare the epitopes obtained from in silico analysis with those obtained from the in vivo experimental investigation in chickens’ vaccination with the recombinant *Salmonella* serovar Heidelberg FlgK protein [84].

## Materials and methods

### Database search for the *Salmonella* serovar Heidelberg FlgK protein

The keywords “*Salmonella* Heidelberg FlgK” were used to search *Salmonella* serovar Heidelberg FlgK proteins in the GenBank database. The amino acid sequences of the *Salmonella* serovar Heidelberg FlgK protein in the FASTA format were downloaded. The MUSCLE alignment software was used for comparison of the FlgK amino acid sequences among a number of *Salmonella *Heidelberg isolates [21, 22]. Molecular mass and pI were calculated by submitting the protein sequence to the ExPASy server [9, 10].

### Analysis of physicochemical characteristics of the FlgK protein

The physicochemical properties (including number of amino acids, molecular mass, theoretical pI, instability index, aliphatic index, and grand average of hydropathicity score) of the FlgK protein was assessed by the ProtParam tool through the ExPASy server [26]. The amino acid sequence of the FlgK protein was directly inputted in the server.

### Linear B-lymphocyte epitope analysis of the FlgK protein

The linear B-cell epitopes of the FlgK protein were predicted by various the B-cell Epitope algorithm tools in the Immune Epitope Database server [76]. These prediction tools included (a) Bepipred Linear Epitope Prediction 2.0 [36], (b) Emini Surface Accessibility, (c) Karplus and Schulz Flexibility, (d) Kolaskar and Tongaonkar Antigenicity, and (e) Parker Hydrophilicity. In addition, SVMTriP [82] was used for analysis of the linear B-lymphocyte epitopes of the FlgK protein.

### Analysis of antigenicity, allergenicity and toxicity of the FlgK protein

The antigenicity of the FlgK protein was analyzed with the VaxiJen (version 2.0) software based on the sequence-independent method for predicting protective antigens [19]. The threshold was set at 0.4 as default, and the accuracy for prediction was 70–89% [19]. The allergenicity of the FlgK protein was predicted by using an AllerTOP (version 2.0) tool based on auto cross covariance to transformation of protein sequences in uniform equal-length vectors [18]. The toxicity of the FlgK protein was analyzed with a ToxinPred tool [29]. All default settings were used for prediction.

### Analysis of physiological properties of the predicted epitopes

The Protein-Sol server was used to predict the physiological properties of the FlgK protein [32].

### Analysis of adhesion of the predicted epitopes

The second generation of Vaxign2 web-based program was used to predict adhesion of the FlgK protein [31, 55]. The default settings were used.

### In vivo animal experiment

Previously, the *Salmonella* Heidelberg FlgK protein was cloned in, expressed in, and purified from *Escherichia coli* cells as described previously [83]. The recombinant protein was emulsified in Freund’s incomplete adjuvant, and the mixture was administered in broilers subcutaneously at one week of age. Each broiler was given 100 µg of the recombinant FlgK protein in a volume of 100 µl. A booster was given at three weeks of age. Blood samples were collected by venipuncture at five weeks of age [84]. Mass spectrometry-based proteomics associated with the immunized chicken sera was used to map the linear immunoepitopes of the recombinant *Salmonella *FlgK protein [84]. Broiler chickens were acquired from a local poultry hatchery at the age of one day, and were raised in isolation units at the density of one square foot per bird in our poultry facilities according to the standard guidelines [79]. Feed and water were provided *ad libitum*. The protocol for the use of chicks in our experiments was approved by the Institutional Animal Care and Use Committee of the U.S. National Poultry Research Center, Agricultural Research Service, U. S. Department of Agriculture, Athens, GA, USA.

## Results and discussion

Vaccination has been regarded as an effective and safe means for controlling infectious diseases in food animal production as well as combatting development of anti-microbial resistance [11, 40, 51]. Commercially available *Salmonella *vaccines for poultry uses are either attenuated or inactivated [15, 52]. However, some disadvantages are associated with uses of attenuated or inactivated vaccines [15, 37, 38, 60]. On the other hand, subunit vaccines that contain key components of a pathogen are able to induce both humoral and cellular immune responses in hosts [2, 16, 60].

The term “*Salmonella* Heidelberg FlgK” was used to search for the amino acid sequences of *Salmonella* serovar Heidelberg FlgK proteins in GenBank. A total of 589 sequences were listed (assessed June 26, 2024), but further examination of each sequence, 289 amino acid sequences were the *Salmonella* serovar Heidelberg FlgK proteins with complete sequences. To determine the similarity of the FlgK proteins among the *Salmonella* serovar Heidelberg isolates, these 289 FlgK protein sequences were subjected to analysis by the MUSCLE alignment software. The results showed that 287 FlgK protein sequences were identical, while two isolates had only one amino acid difference at the position of 239, where the amino acid serine is changed to alanine (Supplementary Fig. 1). The physicochemical properties of the FlgK protein that contains 553 amino acids with calculated molecular mass and pI of 59.11 kDa and 4.79, respectively (Table 1). The calculated aliphatic index, instability index, and GRAVY were 84.34, 30.10 and −0.363, respectively, indicating this protein is thermo-stable in a wide range of temperatures and hydrophilic in nature. When the FlgK proteins from 10 *Salmonella* serovars were compared, the degree of conservation of this protein among serovars is greater than 97%, suggesting that this FlgK protein may be a good vaccine candidate that can induce cross-reactive antibodies against different *Salmonella *serovars [83]. Ten serotypes are *S.* Arizonae, *S.* Typhimurium, *S.* Typhi, *S.* Paratyphi A, *S. *Choleraesuis, *S.* Enderiditis, *S.* Heidelberg, *S.* Schwarzengrund, *S.* Agona and *S. *paratyphi B. Most cause foodborne illnesses in human [65]. Three serotypes are typhoidal types that cause typhoid fever in human [65]. *S.* Choleraesuis is mainly found in swine and can cause severe infection in human [77]. *S. *Arizonae is often found in reptiles [17].

**Table 1:** The physiochemical properties of the *Salmonella* serovar Heidelberg FlgK protein calculated by the ProtParam tool^a^

Physiochemical properties of *Salmonella* serovar Heidelberg FlgK protein	Value
Number of amino acids	553
Molecular mass	59.11 kDaltons
Theoretical pI	4.79
Instability index (II)	30.10 (Stable)
Aliphatic index	84.34
Grand average of hydropathicity (GRAVY)	−0.363

^a^Gasteiger et al. [26]

**Fig. 1:**
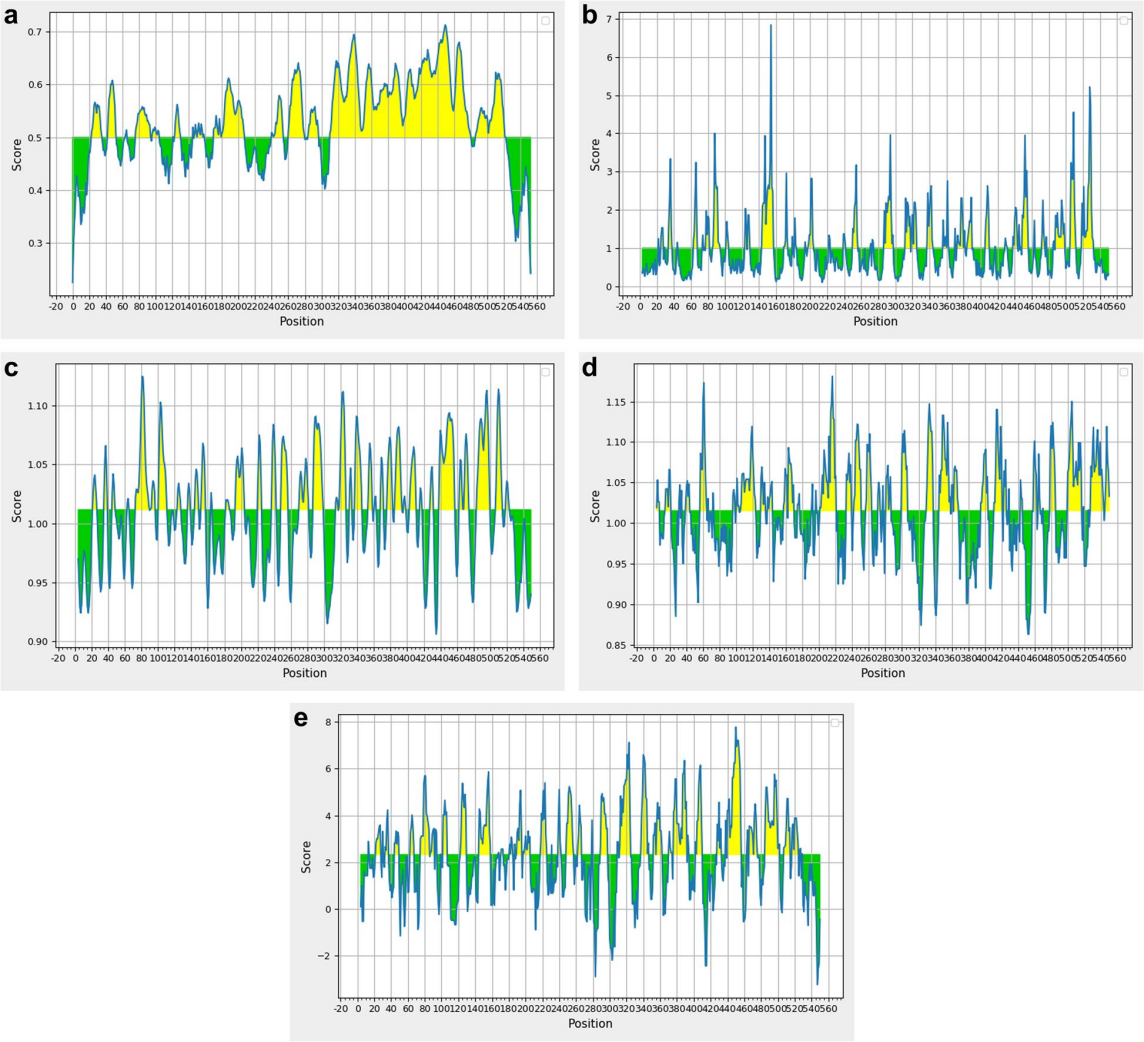
Linear B-lymphocyte epitope prediction of the *Salmonella* serovar Heidelberg FlgK protein. This prediction was carried out through the Immune Epitope Database (IEDB) server (https://www.iedb.org/) [76]. The algorithm methods for analysis included (**a**) Bepipred Linear Epitope Prediction 2.0, **b** Emini Surface Accessibility Prediction, **c** Karplus and Schulz Flexibility, **d** Kolaskar and Tongaonkar Antigenicity, and **e** Parker Hydrophilicity Prediction. The *x*-axis denotes the position of the amino acid residues in the FlgK protein sequence. The *y*-axis indicates the scores assigned to each amino acid residue in the sequence by each method [76]. Color in the yellow areas indicates the amino acid sequences have high probability to be a part of epitopes

Many physicochemical properties of the amino acid residues in the peptide sequences have been used to locate the potential linear continuous epitopes. These properties include flexibility, accessibility, hydrophilicity, surface exposure, turns, helices, and polarity [76]. The FlgK protein sequence of *Salmonella* Heidelberg was analyzed by five B cell linear epitope prediction software as described in the Materials and Methods section. Because each software designs are based on various amino acid properties, the number of potential epitopes in the FlgK protein sequence varied (Fig. 1). For example, the Bepipred software predicted 15 potential epitope peptides, while the Kolaskar and Tongaonkar antigenicity software predicted 18 potential epitope peptides. After carefully comparing these predicted epitopes, there were four almost overlapped consensus epitope sequences, i.e. 77–95, 241–254, 285–297 and 487–522.

Another tool for epitope prediction, VaxiJen v.2.0, was used. The principle of this prediction is solely based on the physicochemical properties of proteins, independent of sequence alignment [19]. This tool identified seven potential epitope peptides in the *Salmonella* serovar Heidelberg FlgK protein at (in the order of scores from high to low): 148–159, 42–53, 358–373, 83–95, 97–105, 475–489 and 171–186.

The results from the Vaxign2 web-based program [31, 55] predicted that this protein had extracellular location (probability = 1.00) and adhesion property (probability = 0.916), indicating this protein is a good candidate for vaccine development. Also, this FlgK protein is probable non-allergen and non-toxin analyzed by the AllerTOP and ToxinPred tools, respectively.

Previously, the epitopes of the *Salmonella *FlgK protein were mapped with mass spectrometry-based immune-capture proteomics using immunized chicken sera [84]. A total of six peptides were recognized by sera from immunized but not the unimmunized chickens at the positions of 77–88, 137–145, 243–255, 358–364, 376–387 and 393–399. Again, after carefully comparing these predicted epitopes with those in vivo experimental investigation with association with the mass spectrometry-based immunoproteomics, three overlapped consensus peptide epitope sequences of the *Salmonella* FlgK protein were identified around the positions of 77–95, 243–255 and 358–373. Minor differences between the predicted and experimental epitope peptides may probably be due to the results of enzymatic digestion during the epitope extraction.

The goal of epitope identification is to use this information for the sub-unit vaccine development against *Salmonella *in broiler chickens. Several methodologies have been used to predict and identify epitopes in proteins, including mass spectrometry-based immunoproteomics and computational algorithms [44, 56, 72]. Previously, *Salmonella *targets for in silico analysis included TolA (membrane-spanning protein), outer membrane proteins (OMPs) and enterotoxins [1, 4, 8, 59, 85], but there are no in vivo experimental data. In our current study, both in silico and in vivo methods were applied to predict the epitopes in the *Salmonella* FlgK protein, which is an important virulence factor in *Salmonella *pathogenesis [6, 27, 66, 69, 80, 81]. Up to 18 epitope peptide sequence were detected by computational methods, while six epitope peptide sequences were obtained from in vivo experimental approach. However, only three epitopes (around the positions of 77–95, 243–255 and 358–373) were detected by both approaches. The discrepancies of these respective approaches may be due to the following factors. For the computational approach, the prediction tools based on various algorithms using the area under the curve are very useful for discriminating the immunogenicity of a protein. Due to not-well understanding of complex, intrinsic interactions between antibody and antigenic epitopes, the thresholds were usually set at the lower than 0.6 that affects the epitope prediction [28, 72]. Therefore, additional experimental data is needed to support the immunoinformatic prediction.

In in vivo experimental study, there are many factors that may affect the epitope mapping. The first question was raised whether the various experimental methods would affect the characterization of the epitope mapping. The epitope extraction method, which an antigen was proteolytically digested with trypsin followed by binding to the antibody and detection by mass spectrometry, was used in our study. Petrotchenko et al. [58] compared multiple techniques, including the epitope extraction, to determine epitopes and found that the experimental results from four approaches agree with each other. Therefore, our approach with the epitope extraction is appropriate. The sites and numbers of a proteolytic enzyme around the epitopes may be the potential limitations of this method.

Next, the quality of antibodies, either monoclonal or polyclonal, is crucial for accurate mapping of epitopes. There are no antibody standards for the *Salmonella *FlgK protein. Broilers were used to produce anti-FlgK antibodies. All sera from immunized but not unimmunized chicks reacted strongly to the FlgK protein in immunoblot analysis [83]. Four and three sera from immunized and unimmunized chicks, respectively, were used for the epitope mapping with the epitope extraction. It is unexpected that three peptides were commonly recognized by both immunized and un-immunized sera. The outcome of the un-immunized serum reactions to these three peptides may be caused by the cross-reactivity to the FlgK protein of other bacteria in the chick gut microbiomes. The sera used in the experiments were not purified so it may affect the binding of peptides and antibodies. We originally expected to have the common shared peptides from four immunized sera, but detected six peptides that were captured by at least one immunized serum. The explanation(s) on no capture of the trypsin-digested peptides by other immunized sera may be due to the affinity of the antibodies in each individual serum sample. It is also possible that genetic variations in chicken immunoglobin repertoires affect their responses to the FlgK protein [47, 64].

In summary, antibodies against *Salmonella* have protective ability in chickens, but they are limited to the homologous serotypes [15]. To develop effective multiple epitope subunit vaccines against *Salmonella* for chicks are highly demanding. In this study, the *Salmonella* FlgK protein was used as an example to predict the epitope peptide sequences by immunoinformatic and immunoproteomic approaches. Our results show that both methods identified three consensus peptide epitope sequences in the *Salmonella* FlgK protein. These findings provide a rational for further evaluation of these shared linear epitopes in vaccine development to cover the chicken population.

## Data Availability

The data and materials in this study are included in this article.
